# Translating nutrition science into national policy: comparative assessment of the 2025–2030 U. S. Dietary Guidelines and the Italian Mediterranean-based guidelines

**DOI:** 10.3389/fnut.2026.1821257

**Published:** 2026-04-21

**Authors:** Laura Rossi, Elena Carrano, Valentina De Cosmi, Rita Di Benedetto, Marco Silano, Umberto Agrimi

**Affiliations:** 1 Department of Food Safety, Nutrition, and Veterinary Public Health, National Institute of Health, Rome, Italy; 2 Department of Cardiovascular and Endocrine-Metabolic Diseases and Aging, National Institute of Health, Rome, Italy

**Keywords:** food-based dietary guidelines, policy translation, protein intake, public health nutrition, sustainability

## Abstract

Dietary guidelines represent foundational instruments of public health governance, influencing clinical practice, food assistance programs, school meal standards, and food system policies. The release of the *Dietary Guidelines for Americans (DGA) 2025–2030* provides an opportunity to reassess how evolving nutrition science is translated into policy in a context characterized by high burdens of obesity and cardiometabolic disease. This review presents a comparative qualitative policy analysis of the 2025–2030 DGA and the Italian Dietary Guidelines for Healthy Eating (2018), aiming to identify structural differences and explore potential policy implications. The analysis examines governance mandates, macronutrient targets, protein sourcing, treatment of ultra-processed foods, alcohol guidance, sustainability integration, and cultural framing. Although both countries draw upon a largely shared body of scientific evidence and converge on core dietary principles, notable divergences emerge in policy emphasis and communication strategies. The DGA 2025–2030 adopt a more prescriptive approach focused on chronic disease prevention, with greater emphasis on protein intake, and explicit reference to ultra-processed foods, while sustainability remains outside the policy scope. In contrast, Italian guidelines are framed within a Mediterranean dietary pattern, emphasize plant-based protein sources and integrate environmental sustainability, food waste reduction, and cultural dimension. These differences may reflect national epidemiological and institutional contexts and variations in policy priorities. This policy review identifies policy trade-offs in guideline development and proposes actionable recommendations to enhance coherence between public health priorities, environmental sustainability, and implementation feasibility in future revisions.

## 1 Introduction

Dietary guidelines represent one of the most influential instruments in public health governance and operate at the intersection of science, policy, economics, and culture (1). Beyond providing recommendations for individual dietary choices, they shape national nutrition strategies, school meal programs, food assistance systems, agricultural incentives, procurement standards, and training of health professional (2). More than 100 countries have developed food-based dietary guidelines (FBDGs), reflecting recognition of diet as a major determinant of non-communicable diseases (NCD) risk. The World Health Organization and the Food and Agriculture Organization have promoted FBDGs as key tools to translate nutrient-based evidence into food-based recommendations accessible to the public (3). However, despite great convergence in scientific understanding, such as the benefits of fruits, vegetables, whole grains, and limited added sugars, national dietary guidelines often diverge in structure and policy scope (4). These divergences reflect variations in the epidemiological burden of disease, which shape the focus of dietary priorities, as well as differences in food system organization and agricultural production patterns that influence what is economically and logistically feasible (5). Cultural dietary traditions affect the acceptability of recommendations and their consistency with established eating patterns (6). Institutional mandates define the scope and authority of guideline development, and political feasibility shapes which measures are adopted (7, 8). Communication strategies further outline how guidance is framed and received by the public, and the extent to which environmental sustainability is integrated or excluded reveals national positioning on the relationship between health policy and ecological responsibility (9). The release of the *Dietary Guidelines for Americans (DGA) 2025–2030* provides a relevant case study. The United States (U. S.) faces persistently high rates of NCDs, contributing substantially to premature mortality and higher health care costs (10). Updated every 5 years, the DGA guide federal nutrition programs and function as a central policy tool (11). In Italy, the Dietary Guidelines for Healthy Eating, last revised in 2018, are grounded in the Mediterranean dietary pattern, a model recognized for cardiometabolic benefits, for cultural heritage and environmental sustainability (12). This policy review examines structural differences between these two guideline systems and considers their implications for countries undergoing metabolic and environmental transitions. The analysis focuses on the policy options outlined in the 2025–2030 U. S. Dietary Guidelines (10) and the Italian guidelines (13), highlighting how shared scientific evidence is translated into distinct policy options under different institutional, cultural, and epidemiological condition.

## 2 Methods

**Figure 1:**
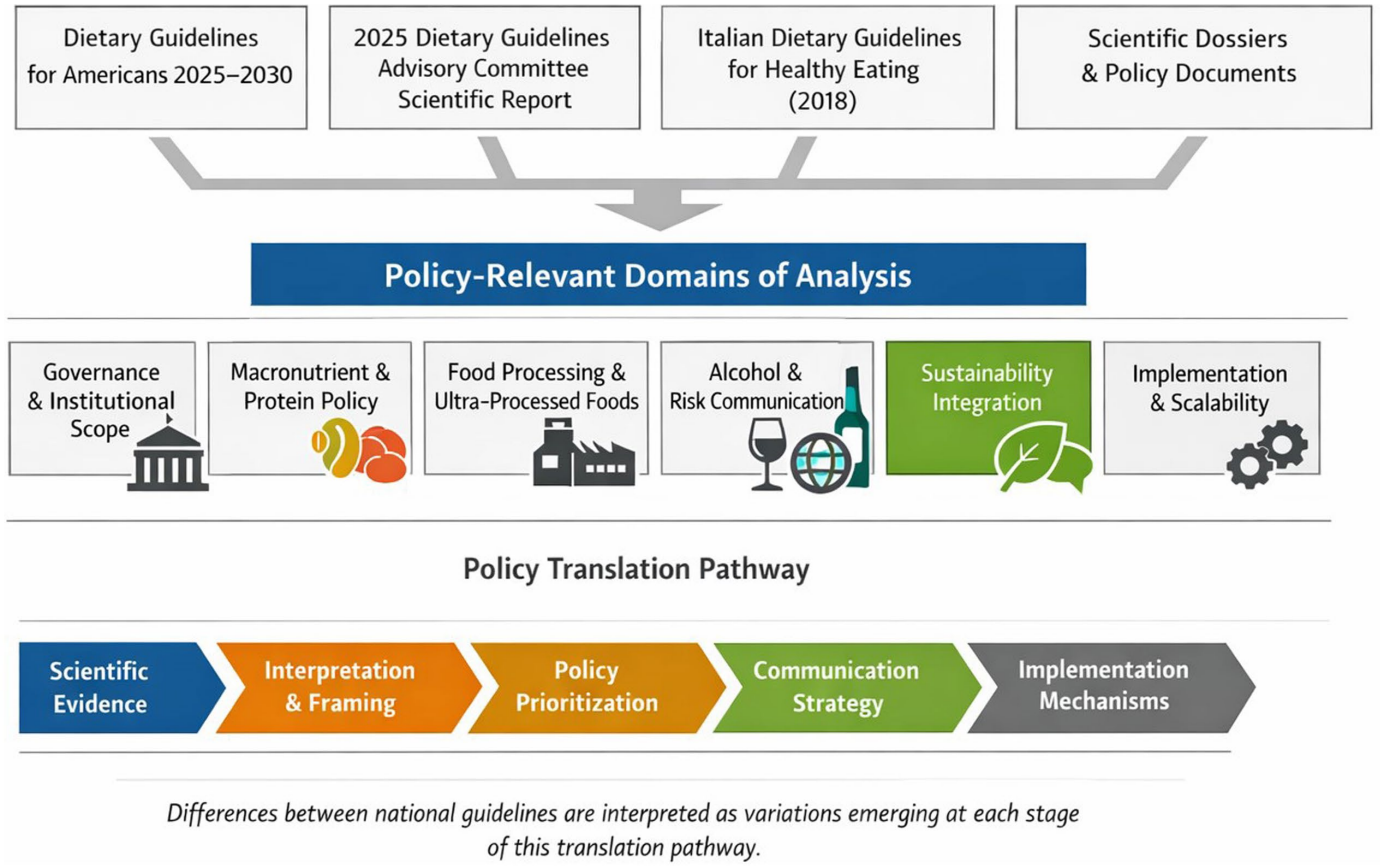
The qualitative policy analysis model comparing US and Italian dietary guidelines.

This study adopts a comparative qualitative policy analysis to systematically examine differences between the U. S. and Italian guidelines (14). The core components of the analytical framework are illustrated in Figure 1. This approach allows structured comparison of policy content, framing, and underlying governance logics, distinguishing it from narrative or commentary-based analyses.

This method is commonly applied in the analysis of national dietary guidelines and in assessing the role of government nutrition policy (15, 16). The analysis focuses on the structural and normative dimensions of guideline formulation. The domains selected for comparison were derived from three complementary criteria: (i) structural relevance within national dietary guideline, (ii) documented variability across high-income countries, and (iii) direct implications for policy implementation and population-level impact. The selected domains are:

1. Governance mandate and institutional scope: dietary guidelines are policy instruments created in a regulatory frameworks, therefore, the assessment of the governance is essential to understand how scientific evidence is translated into advisory guidance (8).
2. Macronutrient distribution and protein policy signaling: recent shifts in protein recommendations constitute strategic signals with both metabolic and environmental implications, functioning as expressions of nutritional guidance and policy orientations (17).
3. Food processing and ultra-processed food framing: the explicit versus implicit treatment of ultra-processed foods (UPFs) represents a relevant aspect of communication strategy, regulatory potential, and alignment with evolving epidemiological evidence (18).
4. Alcohol risk communication: alcohol guidance illustrates how dietary policy navigates tensions between evidence, cultural norms, and political feasibility (19).
5. Sustainability integration: the inclusion, or exclusion, of environmental criteria reflects normative decisions regarding the scope of dietary guidance and the relationship with ecological responsibility (20, 21).
6. Cultural anchoring and communication strategy: cultural framing influences public reception, behavioral adherence, and long-term policy legitimacy (1).
7. Implementation and scalability: to assess how guideline structures interact with institutional systems, food environments, and equity considerations (22, 23).

Each domain was examined through document analysis of:

- Dietary Guidelines for Americans 2025–2030 (10)
- 2025 Dietary Guidelines Advisory Committee Scientific Report (24)
- Italian Dietary Guidelines for Healthy Eating (2018 revision) (13)
- Associated scientific dossiers and policy documents (25)

When findings were reported, distinction was made between content derived from primary guideline documents, scientific advisory reports, and communication materials. Documents were selected based on their relevance as primary national dietary guideline instruments with established public health systems and differing policy traditions. The U. S. and Italy have been selected as contrasting cases due to differences in governance mandates, epidemiological profiles and food systems aspects. The evaluation of the Italian guidelines draws on the same documentary sources as the U. S., even if one of the authors (LR) was directly involved in the Italian guideline development process. This positionality provided additional insights into the formulation of the Italian guidelines but also introduced potential for interpretive bias. To address this, a shared analytical framework was applied systematically across both cases. All authors independently reviewed the materials and engaged in iterative discussions to compare interpretations, with discrepancies resolved through consensus. This process aimed to ensure analytical balance and consistency between the two guideline systems.

The analysis followed a hybrid, deductive-inductive approach. Initial analytical domains were defined deductively based on policy analysis literature and policy translation framework (26). Subsequently, subthemes and interpretative dimensions were refined inductively through iterative engagement with the documents. The analysis was guided by a policy translation framework that conceptualizes dietary guidelines as the product of a multistage process in which scientific evidence is interpreted, and operationalized within institutional and practical constraints (26). Differences between national dietary guidelines were interpreted as variations emerging at different stages of the evidence-to policy translation pathway (27). A comparative matrix is provided as supplementary material (Supplementary Table S1) detailing the structure, operational criteria, extracted elements, and comparative assessment across all analytical domains. Findings were interpreted as reflections of policy framing within the analyzed documents and do not imply causal effects or relationships.

## 3 Assessment of policy and guideline options

**Figure 2:**
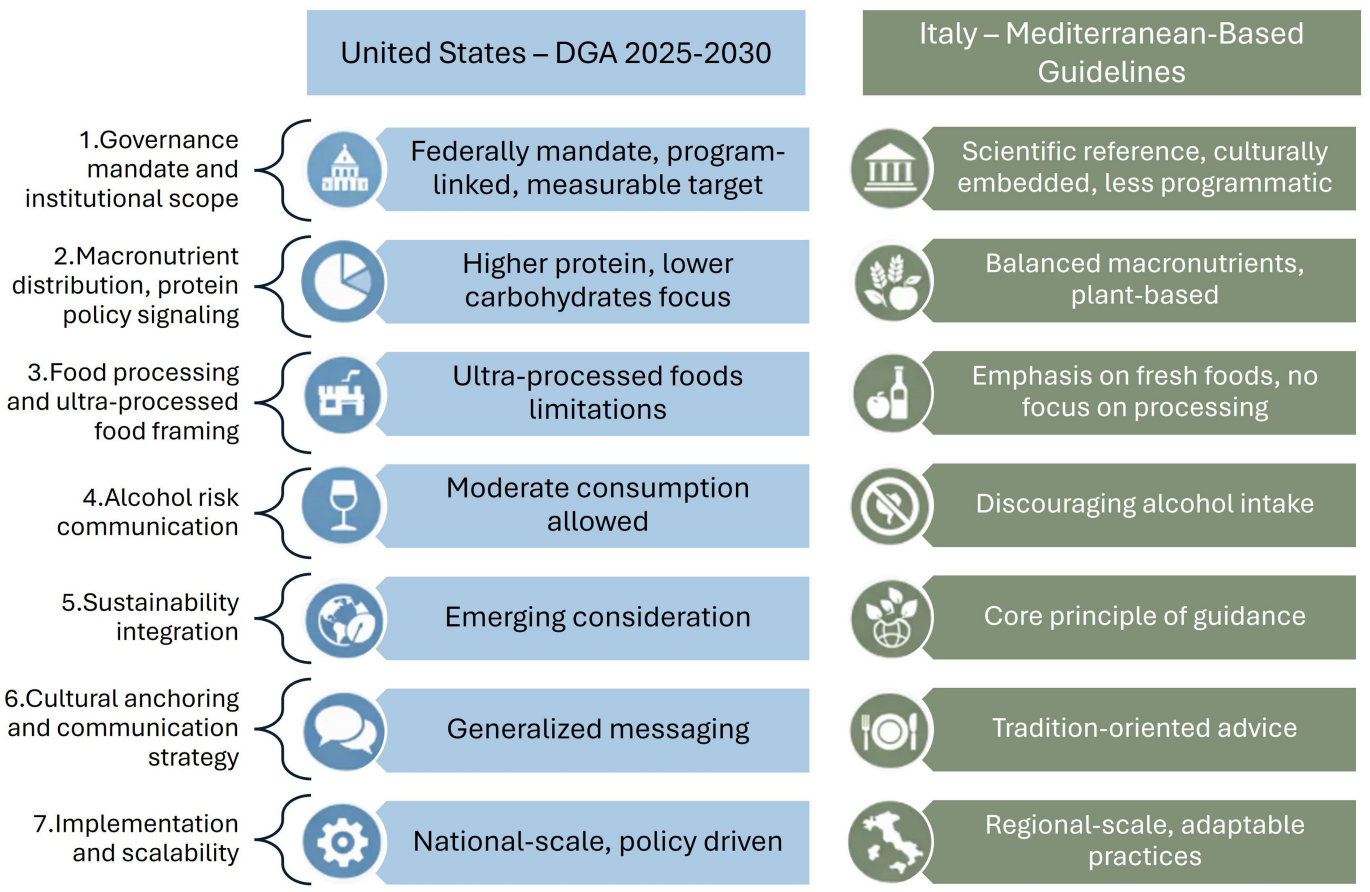
Structural overview of the comparative assessment, highlighting the conceptual positioning of the Dietary Guidelines for Americans 2025–2030 and the Italian Dietary Guidelines systems across the selected domains.

We examined and compared the previously identified seven domains, highlighting areas of convergence and divergence of DGA and Italian Dietary Guidelines and offering a critical reflection on their policy implications. Beyond differences in the content of recommendations, the analysis also highlights how scientific evidence is translated into policy and how recommendations are operationalized within national context. Each domain is presented by distinguishing between (i) observed features of the guidelines, (ii) their possible interpretation within the policy translation framework, and (iii) their policy implications. Unless otherwise specified, references to guideline content refer to primary guideline documents; communication framing elements are explicitly identified when derived from secondary materials. The results of this assessment are summarized in Figure 2.

### 3.1 Governance mandate and institutional scope

The analysis of the guidelines revealed that the DGA are federally mandated under U. S. law and jointly developed by the Departments of Agriculture and Health and Human Services. Their primary objective is to provide guidance to prevent diet-related chronic diseases (28).

The guidelines directly influence:

- Supplemental Nutrition Assistance Program (SNAP)
- National School Lunch Program
- Women, Infants, and Children (WIC) program
- Military and institutional feeding
- Federal procurement standards

In Italy, guideline development is coordinated through scientific research institution (Council for Agricultural Research and Economics-Research Center for Food and Nutrition, CREA-Food and Nutrition) and supported by public health authorities. Although they influence educational materials and policy discourse, they are less directly related to large-scale national programs. The Italian guidelines function more as a scientific reference and educational tool than as a regulatory instrument (12). These findings could be interpreted considering that the integration reflects the role of the DGA as a policy instrument, where recommendations must be compatible with large-scale implementation, budgetary constraints, food supply logistics, and procurement standards. As a result, guideline development in the U. S. is shaped by scientific evidence and by governance priorities, food system dynamics and national policy agendas. From a policy perspective, these factors may influence the feasibility and framing of recommendations, particularly in relation to food availability and institutional scalability. At the same time, consideration of the cultural landscape is essential for ensuring the relevance and adoption of dietary guidance. In highly diverse populations such as the U. S., dietary recommendations must be adaptable to varied cultural practices and socioeconomic contexts, which can affect both communication strategies and real-world adherence.

### 3.2 Macronutrient distribution and protein policy signals

The increased emphasis on protein intake in the DGA (10), supported by the analyses presented in the Dietary Guidelines Advisory Committee Scientific Report (24) emerged as one of the most debated changes. This signals an evolving interpretation of macronutrient priorities compared with previous editions, particularly in relation to aging and metabolic health. In some population groups, recommended intake levels approach approximately 1.2–1.6 g/kg/day, reflecting growing attention to body composition and the prevention of sarcopenia, particularly in aging populations (10). By contrast, the Italian dietary guidelines adopt a more moderate macronutrient philosophy. Protein intake is maintained around 15% of total energy (12), with clear emphasis on legumes as primary protein sources and poultry and fish preferred over red meat. Processed meat is limited to occasional consumption within the broader Mediterranean dietary pattern. Carbohydrates, particularly from whole grains and traditional cereal-based foods, are preserved within a balanced and pattern-oriented framework (12). These features may be interpreted considering that within the U. S. framework, protein is positioned prominently across dietary patterns, with broader inclusion of red meat and greater visibility of dairy products, including whole-fat options as reported in the DGA supporting documents (10, 24). Although carbohydrates remain part of recommended patterns, the emphasis on higher protein intake implicitly supports a relative reduction in refined carbohydrate consumption (29). This approach (24), is claimed to reflect a strategic response to the high prevalence of obesity, insulin resistance, and metabolic syndrome in the U. S. where macronutrient redistribution is viewed as a tool to improve glycaemic control and satiety (24). The observed differences in the framing of protein intake could also be related to the timing of their development within an evolving scientific and policy landscape. Notably, in Italy, the most recent revision of the Dietary Reference Values (30) has increased the recommended protein intake range from 12–18% to 12–20% of total energy intake, while explicitly emphasizing a greater contribution from plant-based protein sources, recognized as protective for both health and environmental sustainability (31). Beyond metabolic considerations, differences in protein recommendations carry environmental implications. Higher consumption of animal-source proteins is associated with increased greenhouse gas emissions, greater land use, and higher water demand compared with plant-based alternatives (32). In this sense, macronutrient distribution may reflect larger policy trade-offs between short-term metabolic objectives and long-term sustainability considerations.

### 3.3 Food processing and ultra-processed foods framing

One observed aspect explicitly reflected in the Dietary Guidelines for Americans 2025–2030 (10) is the acknowledgment of UPFs as a category of concern. While previous editions emphasized limiting added sugars, sodium, and saturated fats, the current guidelines move further by identifying highly processed foods as structurally linked to poor dietary quality (33). This shift aligns with a growing body of epidemiological literature associating UPFs consumption with obesity, cardiovascular disease, type 2 diabetes, and all-cause mortality (34). The Italian dietary guidelines (13) adopt a more implicit approach. They do not use the formal language of UPFs, they emphasize limiting foods high in added sugars, salt, and saturated fats and recommend prioritizing fresh, seasonal, and minimally transformed products.

The explicit positioning of UPFs in the DGA could be interpreted as having both strengths and potential limitations. On the one hand, the clarity of discouraging UPFs may enhance public understanding and improve communication coherence (18). This framing (10), simplifies complex nutritional advice into a more intuitive directive: prioritize minimally processed, nutrient-dense foods. On the other hand, the operationalization of the ultra-processed category remains contested. The NOVA classification system (35) has been criticized for potential heterogeneity within categories and for not always distinguishing between processing that preserves safety and processing that introduces harmful additives or hyper-palatable formulations (36). From a policy perspective, these divergent approaches illustrate two distinct strategies for addressing industrial food environments. The U. S. model directly confronts processing as a structural determinant of diet quality, potentially paving the way for regulatory measures and labeling reforms. By contrast, the Italian model relies more on cultural and culinary norms to guide behavior, placing greater emphasis on nutritional profiling than on the degree of industrial processing. As food systems continue to globalize, and the availability of UPFs expands in European markets, the effectiveness of more implicit approaches may require reassessment (37, 38). In this context, clear front-of-pack labeling (FoPL) plays a key role in supporting more informed and healthier choices (39). Evidence from several countries shows that FoPL policies can influence consumer behavior and encourage product reformulation (40). For example, Chile (41) and Mexico (42) introduced mandatory warning labels on foods high in sugar, salt, calories, or saturated fats. In Europe, France implemented the Nutri-Score (43) and the United Kingdom developed the traffic light labeling scheme (44); in Italy, the “NutrInform Battery” represents a different approach, providing information on the contribution of a portion of food to daily nutrient intake through a battery-style visual format (45).

### 3.4 Alcohol risk communication

The analysis highlighted that alcohol guidance is another area of divergence. Although wine historically occupies a place within Mediterranean culinary culture, the current guidelines clearly state that alcohol is harmful for health and should not be promoted as protective (13, 46, 47). This position reflects evolving evidence demonstrating that even low levels of alcohol consumption carry measurable carcinogenic risk and that previous assumptions regarding cardiovascular benefits are increasingly questioned (48).

The guidance on alcoholic beverages and health in the DGA is informed by the National Academies of Sciences, Engineering, and Medicine report (19) although the final Guidelines text reflects only partial integration of its conclusions (10). The U. S. DGA adopt a cautious but less categorical stance, recommending limiting alcohol consumption and advising that individuals who do not drink should not begin drinking for health reasons. However, moderate consumption remains within the scope of permissible behavior (10).

The divergence between these approaches needs to be interpreted considering the complexity of alcohol risk communication in dietary policy: while categorical exclusion may enhance clarity, moderated messaging may improve feasibility but risk ambiguity. However, from a public health perspective, explicitly acknowledging that no level of alcohol consumption is entirely risk-free is in line with health protective approaches and strengthen transparency (49). These differences highlight how dietary guidelines function as instruments of social negotiation as much as scientific documents. In many countries, adoption of alcohol control initiatives is hindered by the alcohol industry’s strong lobbying (50, 51). Evidence shows that industry relies on recurrent tactics across contexts: building long-term relationships with policymakers, promoting “individual responsibility” narratives, supporting targeted education initiatives, advocating for self- or co-regulation, and positioning themselves as legitimate partners in prevention (52).

### 3.5 Sustainability integration

The outcomes related to sustainability, indicate that the Italian dietary guidelines explicitly integrate sustainability principles avoiding framing them as an external obligation. These guidelines recognize that dietary choices influence environmental outcomes through greenhouse gas emissions, land use, water consumption, and biodiversity impact (53, 54). Recommendations include prioritizing seasonal and local foods, reducing red meat consumption, minimizing food waste, and preserving culinary traditions that align with lower environmental footprints. The Mediterranean diet is framed as cardioprotective and as ecologically sustainable (47). In the DGA 2025–2030 the environmental issues remain outside the formal scope of the guidelines themselves, even if it may appear in broader federal policy discussions (10). This exclusion is historically rooted. Previous attempts to incorporate sustainability into DGA encountered political resistance, reflecting tensions between agricultural interests, environmental policy, and public health mandates (55). The differences observed between the U. S. and Italian guidelines can be interpreted within the context of dual global transitions: the rising obesity and NCDs prevalence, and the climate change and ecological degradation. High-income countries face the challenge of designing dietary policies that simultaneously address both domains (56). The U. S. DGA respond primarily to the metabolic crisis, emphasizing macronutrient redistribution and UPFs reduction. The Italian guidelines respond to metabolic concerns while also considering environmental sustainability within the Mediterranean model. These strategies reflect differing national priorities and illustrate potential pathways for integration.

The omission of sustainability may have important policy implications. Diet-related environmental impacts are increasingly recognized as central to planetary health (20). Livestock production contributes significantly to greenhouse gas emissions and resource use. Integrating sustainability metrics into dietary guidelines can align nutrition policy with climate mitigation strategies and sustainable development goals (21). However, expanding guideline scope also increases political complexity and may challenge existing agricultural systems. An emerging question for future policy development is whether sustainability considerations can be systematically incorporated into dietary guidance without compromising clarity or feasibility (57). As scientific consensus grows around the environmental impacts of dietary patterns, exclusion of sustainability from national guidelines may become increasingly difficult to justify. At the same time, integrating environmental criteria must avoid overcomplicating public messaging or imposing unrealistic behavioral expectations (58). From these perspectives, dietary guidelines are instruments of environmental health action. Two conceptual frameworks, the exposome and human–environment symbiosis offer useful insights for policy innovation. The exposome highlights how lifelong exposure to environmental factors interacts with biological processes to shape health outcomes (59). Similarly, the concept of symbiosis emphasizes the interdependence between human health and ecological systems, reinforcing the need for dietary patterns that sustain both (60). Considering these aspects, dietary guidelines do not represent isolated behaviors but part of a complex system linking food environments, lifestyle factors, and disease risk. UPFs are often associated with resource-intensive production, extensive packaging, and long supply chains (61), conversely, traditional dietary pattern, such as the Mediterranean diet, offer a model of alignment between cultural heritage, nutritional quality, and environmental sustainability (62). Integrating these dimensions into dietary guidance may therefore support more resilient food policies, narrowing the gap between metabolic health, environmental sustainability, and cultural value.

### 3.6 Cultural anchoring and communication strategy

The documents’ analysis highlighted that the Italian Dietary Guidelines are explicitly rooted in the Mediterranean diet. By linking health recommendations to traditional foods, culinary practices, seasonal eating, and convivial social norms, the Italian framework leverages collective identity as a mechanism to reinforce adherence and legitimacy. This culturally grounded approach allows dietary advice to be presented as continuity rather than change, thereby reducing resistance and enhancing behavioral feasibility (47). In contrast, the DGA operate within a highly diverse and heterogeneous sociocultural context, which limits the feasibility of anchoring recommendations in a single culinary tradition. These differences may be interpreted considering distinct sociocultural contexts, which shape the content of dietary guidelines, their narrative structure and public reception.

From a policy perspective, this context helps explain why the DGA adopt a more universal and health-centered communication strategy, emphasizing nutrient quality food groups, and the reduction of UPFs. This is reflected in broadly applicable messaging, including expressions such as “eat real food,” derived more from associated public communication materials linked to the DGA (29) rather than from the guideline text itself.

### 3.7 Implementation and scalability

The documents’ analysis revealed that the DGA are closely tied to federal nutrition programs. This structural integration necessitates clarity and measurable targets. Recommendations must be compatible with large-scale procurement systems, budgetary constraints, and food supply logistics (10). As a result, the DGA tend to prioritize quantifiable nutrient thresholds and food groups distributions that can be translated into standardized menu planning and labeling frameworks.

The Italian dietary guidelines are less directly linked to centralized feeding programs of comparable scale. Their implementation relies more heavily on public health campaigns, educational materials through schools, media, and community initiatives (12). This context allows for a more narrative and culturally rooted communication style, emphasizing moderation, conviviality, and culinary tradition. From a implementation perspective, both systems illustrate the importance of aligning guideline recommendations with structural determinants of food choice.

These findings should be interpreted considering that the ultimate impact of dietary guidelines depends on their translation into practice across multiple institutional and social settings (63). Guidelines function within complex implementation ecosystems that include health care systems, educational institutions, public procurement mechanisms, and mass communication strategies (15). Consequently, differences in policy framing between the U. S. and Italy must also be considered through the lens of implementation and institutional integration. For example, the effectiveness of recommendations targeting UPFs should be interpreted in relation to food environments (64). In the U. S., UPFs are widely available across public and commercial settings, including schools, workplaces, and retail environments, and are heavily promoted through marketing strategies that shape consumer preferences and purchasing behaviors (65). At the same time, economic constraints play a critical role. Energy-dense, highly processed foods are often more affordable and accessible than fresh and minimally processed alternatives, particularly in low-income communities (66). This cost differential, combined with structural inequalities in food access, may limit the feasibility of adhering to guideline recommendations, highlighting the importance of aligning dietary guidance with policies addressing affordability and food environments.

From a policy perspective, dietary guidelines should explicitly address equity implication aspects, given that diet-related chronic diseases disproportionately affect socioeconomically disadvantaged populations (67). Higher-protein diets may be economically inaccessible to low-income populations and may increase financial burden if not accompanied by supportive food policies (68). Conversely, plant-based dietary patterns can offer cost-effective pathways to nutritional adequacy but require culinary skills and time resources that are not evenly distributed across socioeconomic strata (69). In the U. S., structural inequities in food access remain significant. Communities with limited access to fresh products and with high exposure to UPFs may find guideline adherence challenging (70). While the DGA articulate nutrient-based targets and encourage minimally processed foods, broader policy alignment with agricultural subsidies and food marketing regulation remains essential for equitable implementation. In Italy, socioeconomic disparities in dietary quality are also emerging, particularly among younger generations and lower-income groups (71). Economic pressures and globalized food marketing have shifted consumption patterns toward a more westernized diet (72, 73). In the Italian guidelines, the affordability aspect is addressed with practical advice on how to have a nutritious diet with low-cost foods emphasizing plant-based dietary components (13). Equity considerations also intersect with alcohol guidance and UPFs messaging. Vulnerable populations are often more exposed to aggressive marketing of unhealthy foods and alcoholic beverages (74). Clear, consistent, and evidence-based communication strategies are therefore critical for reducing health disparities.

## 4 Actionable recommendations

**Figure 3:**
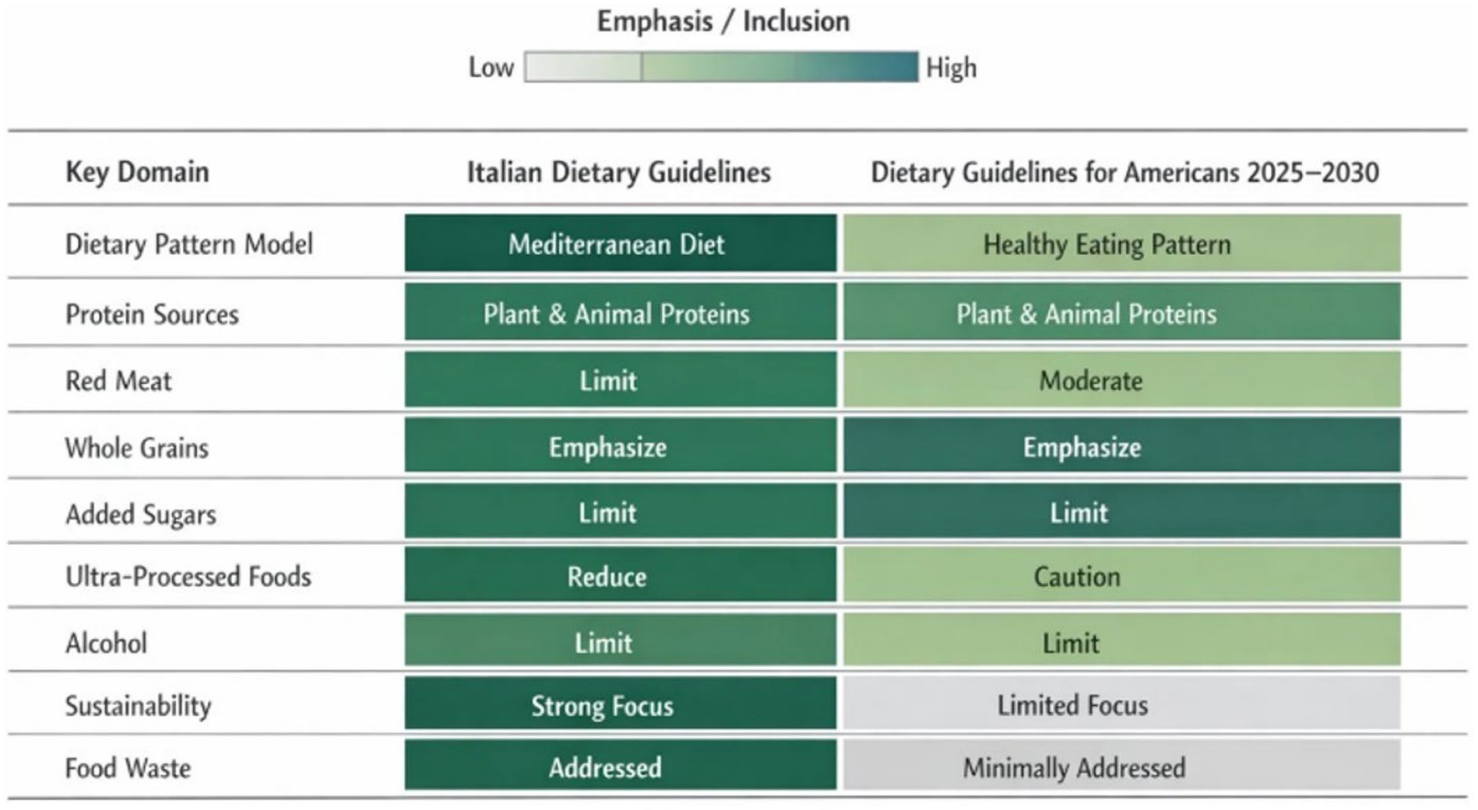
Comparative matrix displaying the inclusion and degree of emphasis placed on nutrition and sustainability dimensions, identifying areas of convergence and divergence between the Dietary Guidelines for Americans 2025–2030 and the Italian Dietary Guidelines.

The comparative assessment of the DGA and the Italian guidelines highlight distinct approaches to policy translation. Figure 3 presents a comparative matrix that synthesizes the relative emphasis and inclusion of key nutrition and sustainability domains within each framework. The categories of relative emphasis (e.g., stronger vs. more limited emphasis) shown in the figure should not be interpreted as quantitative scores. Rather, they are derived from a structured qualitative process applied consistently across both guideline systems. The observed divergences underlined strategic priorities and governance choices highlighting areas where future guideline revisions could be further strengthened. The following recommendations build on both the structural analysis (Supplementary Table S1) and the comparative matrix illustrated in Figure 3, with the aim of enhancing coherence, transparency, and the long-term effectiveness of national dietary guidelines as key instruments of public health policy. They are directed to specific institutional actors and propose actionable mechanisms informed by existing international experiences.

### 4.1 Recommendation 1—enhancing transparency in evidence-to-policy translation

Future dietary guidelines should more clearly distinguish between recommendations based on scientific evidence, those derived from expert interpretation, and those influenced by institutional mandates. These aspects should be explicitly reported in the guidelines supporting documents.

### 4.2 Recommendation 2—integrating sustainability as a core dimension of dietary guidance

Environmental sustainability should be incorporated alongside nutritional adequacy and health outcomes, particularly in high-income settings. Collaboration with ministries of health, agriculture, and environmental agencies should be foreseen using standardized indicators such as greenhouse gas emissions, land use, and water use adapted to national contexts. Countries such as Brazil, Canada, European Nordic Countries, as well as France, Spain, Italy. have already incorporated sustainability principles into their dietary guidelines, demonstrating the feasibility of this approach and its potential to align public health and environmental objectives (57).

### 4.3 Recommendation 3—contextualizing macronutrient targets within health and environmental goals

Macronutrient targets should be defined within health and sustainability objectives. Particularly, proteins recommendations should be tailored by advisory committees and public health authorities according to age, activity level, and clinical need, whereas public guidance should emphasize consuming plant-based protein sources. This can be operationalized through food-based dietary recommendations and procurement standards, supporting alignment between nutritional targets, chronic disease prevention, and environmental sustainability.

### 4.4 Recommendation 4—refining communication strategies around UPFs

The explicit acknowledgment of UPFs in the U. S. DGA 2025–2030 (10) further contextualized in supporting scientific literature (75) and advisory reports (24) represents a significant advance in addressing modern food environments. Public health authorities and ministries of health should provide practical explanatory tools that distinguish between minimally processed foods, processed culinary ingredients, and industrial formulations linked to whose consumption is linked to a higher risk of NCDs. These distinctions should be supported through consumer education materials, visual tools, and professional training, helping avoid oversimplification while improving food literacy. Lessons from countries such as Brazil; several Latin American nations (including Chile, Ecuador, Mexico, Peru, and Uruguay); Canada; as well as France and Portugal, where dietary guidance and labeling policies have incorporated these concepts, should be considered, given their contribution to improving consumer awareness and supporting healthier food choices (18).

### 4.5 Recommendation 5—strengthening alcohol risk communication

Future dietary guidelines should adopt transparent and consistent messaging on alcohol related risks. Explicit acknowledgment that no level of alcohol consumption is entirely without risk and is not recommended nor advised, may improve alignment with scientific consensus. This approach should be led by ministries of health and public health agencies, and reinforced through targeted communication campaigns and labeling policies, particularly for vulnerable groups. Evidence from several countries suggests that clearer risk communication can improve awareness and support healthier consumption patterns (76).

### 4.6 Recommendation 6—cultural adaptation in implementation planning

Cultural relevance is essential for adherence and long-term sustainability of dietary patterns. Implementation should be supported through region-specific and culturally adapted educational materials, involving public institutions, schools, community organizations, and media actors. Countries such as Italy (77) and Mexico (78) demonstrate how embedding dietary guidance within traditional food cultures can enhance both acceptability and sustainability of dietary recommendations.

### 4.7 Recommendation 7—promoting cross-national dialog and policy learning

Structured international exchange can strengthen guideline development. Many countries face shared challenges related to food system transformation, and environmental sustainability. Establishing formal mechanisms for cross-national exchange between guideline advisory committees could facilitate harmonization of core scientific principles while preserving contextual flexibility. International organizations and academic consortia can facilitate this process by promoting methodological transparency and shared evaluation approaches.

## 5 Discussion

This comparative analysis of the DGA and the Italian Dietary Guidelines illustrates a transformation occurring within global nutrition policy. Dietary guidelines are increasingly operating at the intersection of metabolic health, environmental sustainability, cultural identity, and food system governance. The divergences observed between the U. S. and Italy do not reflect contradictions in scientific evidence, but alternative pathways for navigating the policy terrain. At their core, dietary guidelines are governance instruments. They structure public understanding of healthful eating, inform institutional procurement, and indirectly impact agricultural demand. As such, their evolution mirrors societal priorities (1, 4). The DGA, emerging in a context of severe cardiometabolic burden, emphasize metabolic risk mitigation through clearer macronutrient signaling and explicit discouragement of UPFs. This approach appears to prioritize measurable health outcomes and operational feasibility within large-scale federal programs (33). The Italian guidelines, by contrast, demonstrate how dietary policy can embed cultural continuity and environmental stewardship alongside health promotion. The Mediterranean model is a sociocultural pattern linking food, identity, conviviality, and sustainability. This integrative framing expands the scope of dietary guidance beyond metabolic endpoints (12).

Cultural dietary traditions are determinants of food practices and influence long-term health of populations, with implications for both the prevention of NCDs and the development of nutrition-related conditions (79). While these relationships are complex and not deterministic, they underscore the importance of considering population-specific biological and cultural contexts in dietary guidance. Adherence to culturally grounded dietary patterns may support metabolic health through their nutritional composition and alignment with established food practices, thereby enhancing feasibility and the long-term sustainability of dietary behaviors (80). These distinct trajectories reveal that guideline development reflects normative decisions about scope and responsibility (81). The present paper shows that decisions such as incorporating environmental recommendations, or prioritizing prescriptive thresholds over pattern-based flexibility, are governance choices as much as scientific ones.

The DGA appear to respond primarily to the metabolic dimension of the dual burden, defined as the coexistence of both NCDs and environmental crisis. By emphasizing protein intake and UPFs reduction, they seek to recalibrate macronutrient distribution and improve diet quality in a population with high prevalence of obesity and NCDs (10). However, the relative absence of sustainability considerations may limit the capacity of the guidelines to align with climate mitigation strategies. However, while the recommendation of increasing protein intake may offer benefits in specific contexts, this approach also warrants consideration of potential health implications. Higher intake of animal-source proteins, particularly red and processed meats, has been associated with increased risk of certain cancers (82). Moreover, the applicability of higher-protein dietary patterns should be interpreted with caution across different populations. Dietary responses may vary according to cultural dietary patterns, baseline nutritional status, and biological and genetic factors influencing metabolism (83). As such, recommendations emphasizing increased protein intake should be contextualized within population-specific health profiles and dietary traditions, rather than adopted uniformly.

The Italian guidelines illustrate how dietary policy may be combined with sustainability aspects. However, environmental recommendations were largely developed through expert consensus rather than a structured quantitative data framework. The advantage of the presence of the Mediterranean model facilitates the development of guidelines with sustainability language better accepted in terms of agricultural policy, consumer behavior, and food system resilience (84). The comparative findings therefore suggest that future dietary guideline revisions may need to address health and ecological objectives simultaneously rather than sequentially. Integrating environmental metrics does not necessarily dilute health messaging; instead, it may strengthen policy coherence and long-term credibility (53). Another central dimension emerging from the comparison concerns risk communication. Clear messaging around UPFs and alcohol reflects evolving evidence but also requires careful calibration to avoid oversimplification. Public trust in dietary guidelines depends on perceived transparency and consistency (85); thus, clarifying that alcohol carries risk at any consumption level aligns communication with scientific consensus (48). On the other hand, presenting protein targets as responsive to specific demographic needs rather than universally optimal may prevent misinterpretation (86).

Thus, strengthening clarity in the evidence-to-policy pathway is a public health imperative (87). An important dimension of this comparison concerns the temporal gap between the two guideline systems. The U. S. Dietary Guidelines 2025–2030 reflect more recent scientific evidence and policy developments, whereas the Italian guidelines are grounded in the state of knowledge and policy priorities as of 2018. Some of the observed divergences, including the explicit treatment of UPFs (75) and the framing of protein intake (88, 89) may therefore partly reflect broader shifts in scientific consensus and policy discourse over time, rather than solely differences in national strategies. Accordingly, the findings should be interpreted through both contextual (country-specific) and temporal (evolution of evidence and policy) lenses, recognizing that dietary guidelines are dynamic instruments shaped by the scientific advancements and consequent policy development (4).

The findings of this analysis demonstrate that realistic considerations based on best practice are essential in dietary policy, as recommendations that fail to account for real-world constraints risk limited adoption and impact. Recommendations that are nutritionally optimal but economically or logistically inaccessible may widen health disparities. Integrating affordability analyses and considering time constraints, and food access inequalities can enhance equity-oriented policy design (90).

The divergences between the DGA and Italian guidelines illustrate adaptive responses to differing national constraints and priorities showing the importance of country-specific nutrition recommendations (91). The U. S. model demonstrates how guidelines can directly confront industrial food environments and metabolic risk. The Italian model demonstrates how health and sustainability can be integrated within a culturally resonant dietary pattern. The divergences observed between the U. S. and Italian guidelines are not isolated phenomena but part of a wider international landscape of dietary policy evolution (92). Several countries, including Canada, Brazil, Mexico, and Nordic nations, have adopted explicit references to food processing and sustainability in their guidelines. The Brazilian dietary guidelines, for example, strongly emphasize avoidance of UPFs and protection of traditional food culture (93). Similarly, Mexico’s recent dietary guidelines (78) incorporate sustainability principles and draw on traditional food systems such as the *milpa,* a dietary pattern based on maize, beans, and squash, reflecting a culturally grounded approach comparable to the Mediterranean model underpinning the Italian guidelines. Canada’s Food Guide integrates plant-based emphasis and sustainability considerations (94). European Nordic countries’ guidelines incorporate environmental impact assessments and climate targets (95). Within this global context, the Italian approach aligns more closely with European and Latin American models. The U. S. approach remains more tightly focused on metabolic health and nutrient adequacy.

National dietary guidelines must remain adaptable to local production systems, culinary traditions, and public health priorities (96). Cross-national dialog mechanism should facilitate knowledge exchange while respecting contextual differences.

The analysis reported in this paper contributes to a growing literature on the evolution of FBDGs as multidimensional policy instruments. It underscores the need for transparent decision-making processes that clarify how evidence, feasibility, and political constraints contribute to the development of the final recommendations. Ultimately, dietary guidelines must remain dynamic instruments capable of responding to scientific advances, demographic shifts, and environmental constraints. The credibility of future revisions will depend on their ability to integrate multiple dimensions of health without sacrificing clarity or feasibility.

### 5.1 Strengths and limitations

Several strengths enhance the robustness and relevance of the analysis. First, this review adopts an explicit qualitative document-based policy analysis, examining the content of recommendations and the processes through which scientific evidence is interpreted and operationalized. By conceptualizing dietary guidelines as policy instruments rather than static scientific documents, the analysis captures structural dimensions often overlooked in purely nutritional comparisons. This perspective allows for identification of trade-offs embedded within guideline development, including possible competing priorities between metabolic health priorities, environmental sustainability, and political feasibility. Second, the comparison focuses on two high-income countries with established public health infrastructures and documented chronic disease burdens, making the findings particularly relevant to other countries undergoing similar nutritional transitions. The contrast between a federally mandated, programmatically integrated guideline system (U. S) with a culturally rooted, sustainability-integrated Mediterranean model (Italy) provides analytical analysis showing alternative pathways for translating nutrition science into policy. Third, the present work situates national divergences within a wider international landscape, recognizing that dietary guideline evolution is influenced by global scientific debate and planetary health considerations. This contextualization enhances the external relevance of the findings and supports the development of actionable recommendations applicable beyond the two countries examined.

The paper shows also limitations that warrant consideration. The analysis of DGA is based on qualitative document review of publicly available guideline texts and related scientific reports. As a qualitative document-based analysis, this study focuses on policy content, framing, and the translation of scientific evidence into guideline recommendations. It does not assess implementation processes, stakeholder dynamics, or real-world outcomes. Accordingly, the findings should be interpreted as insights into policy design and evidence-to-policy translation rather than as evidence of causal effects on population health. While this approach is appropriate for examining policy structures, it does not capture implementation fidelity, population adherence, or health impacts associated with each guideline system. Therefore, observed differences in policy framing may not necessarily translate into measurable differences in dietary behaviors or disease outcomes. The evaluation of the Italian guidelines draws on the same documentary sources as the U. S. case; however, one of the authors (LR) was directly involved in the Italian guideline development process. This positionality may introduce potential interpretative bias and reflexivity issues. To mitigate this, all authors engaged in iterative analytical discussions, and discrepancies in interpretation were critically examined and resolved through consensus. This process aimed to enhance analytical balance and reduce the influence of individual perspectives on comparative analysis. Additionally, the comparison is limited to two national contexts. While these cases provide meaningful contrast, they cannot fully capture the diversity of approaches observed globally. Another limitation concerns the dynamic nature of nutrition science. Research continues to evolve, particularly regarding UPFs, protein requirements, and alcohol-related risk. The DGA were published approximately 6 years after the latest revision of the Italian guidelines, and this temporal gap affects the comparability of the two documents. Some observed differences, particularly regarding UPFs and protein recommendations, may reflect evolving scientific evidence and policy debates over time. In contrast, differences in other domains, such as alcohol and sustainability, appear less attributable to temporal factors and may instead reflect distinct policy priorities and governance approaches, including the relative weighting of public health considerations within larger policy frameworks. Finally, political and economic constraints influencing guideline development are not always explicitly documented in official texts. As a result, inferences regarding feasibility considerations or stakeholder influence are necessarily speculative. While grounded in policy analysis principles, such interpretations may not capture the full complexity of internal deliberations within advisory committees.

## 6 Conclusion

**Figure 4:**
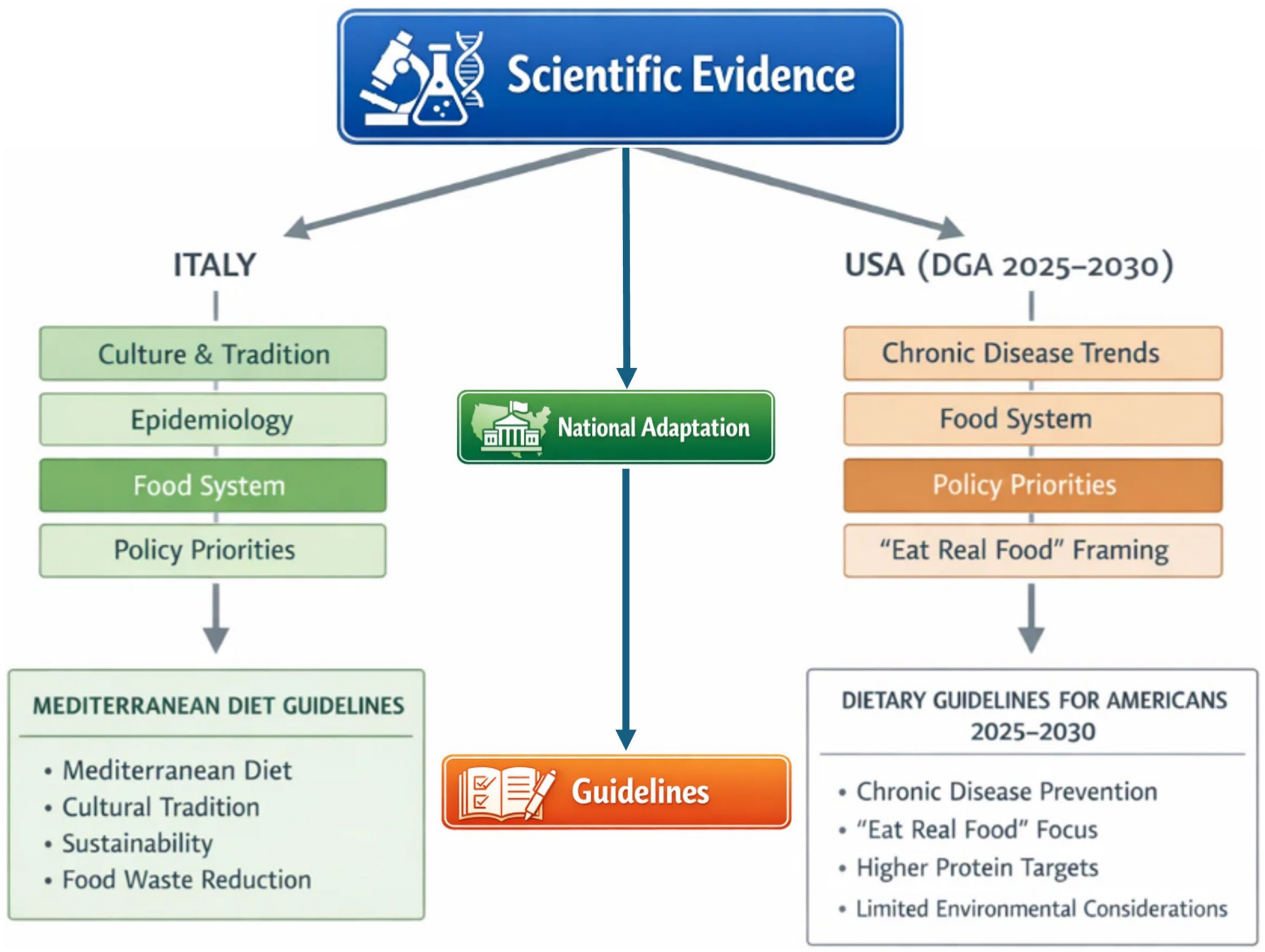
Conceptual schematic model of national adaptation in dietary guideline development.

This comparative analysis highlights that dietary guidelines are dynamic governance instruments shaped by national epidemiology, institutional mandates, sociocultural contexts, and political feasibility. Although both the U. S. and Italian guidelines draw on a broadly shared scientific evidence base, their translation into policy reflects distinct priorities and normative orientations. Figure 4 illustrates how common scientific foundations are filtered through country-specific factors, including disease burden, food system structure, cultural identity, and policy mandates, leading to differentiated policy outputs.

The findings of this work suggest that future dietary guideline development may benefit from more transparent decision-making processes that articulate the steps connecting scientific evidence to policy recommendations. In addition to that, this study highlights the need for countries to move beyond one-size-fits-all approaches and instead develop food policies that are both culturally grounded and biologically appropriate for their populations. Dietary guidelines and related policy instruments should reflect local food environments, culinary traditions, and epidemiological profiles, while also incorporating emerging evidence on nutrition, health, and sustainability. This includes promoting dietary patterns that support environmental resilience and policies that both address nutritional adequacy and consider ecological impacts, fostering health-promoting and sustainable food systems. These processes may be influenced by commercial actors whose interests may conflict with public health objectives. The establishment of clear governance mechanisms that ensure transparency and manage conflicts of interest, prioritizing equity sustainability, and implementation are crucial elements for future guideline development.
