# Supplementary material for: Translating nutrition science into national policy: comparative assessment of the 2025–2030 U. S. Dietary Guidelines and the Italian Mediterranean-based guidelines
Source: Front Nutr. 2026 Apr 21;13:1821257. doi: 10.3389/fnut.2026.1821257 (PMC13139003; doi:10.3389/fnut.2026.1821257)
Supplement: Supplementary file 1 [file Table_1.docx]

Supplementary Material

Supplementary Table 1. Coding framework and comparative matrix used for the qualitative document-based policy analysis of U.S. and Italian dietary guidelines.

| **Domain** | **Operational Criteria** | **Key indicator/Guiding questions** | **U.S. DGA 2025–2030 (evidence extracted)** | **Italian Guidelines 2018 (evidence extracted)** | **Coding outcome** | **Comparative assessment** |
| --- | --- | --- | --- | --- | --- | --- |
| **Governance mandate and institutional scope** | Extent to which dietary guidelines are legally mandated and integrated into national policy instruments and programs | *Are guidelines legally mandated? Are they linked to national programs (e.g., school meals, food assistance)? Do they have regulatory or operational functions?* | Federally mandated; jointly issued by USDA and HHS; directly linked to SNAP, WIC, school meals, military feeding, and procurement standards | Developed by CREA; advisory and educational role; not directly embedded in large-scale national feeding programs | **High institutional integration (U.S.) vs Moderate integration (Italy)** | **U.S. guidelines function as operational policy instruments; Italian guidelines primarily serve as scientific and educational references** |
| **Macronutrient distribution and protein policy signaling** | Extent to which macronutrient targets, particularly protein, are explicitly quantified and framed as policy priorities | *Are quantitative intake targets provided? Is protein intake emphasized? What sources (animal vs plant) are promoted?* | Increased emphasis on protein intake (up to ~1.2–1.6 g/kg/day in specific groups); inclusion of animal-source proteins, including red meat and dairy | Moderate protein (~15% energy); emphasis on legumes and plant-based sources; limited red meat within Mediterranean pattern | **High quantitative emphasis (U.S.) vs Moderate pattern-based emphasis (Italy)** | **U.S. guidelines place stronger emphasis on protein quantity and metabolic outcomes; Italian guidelines emphasize dietary patterns and plant-oriented sources** |
| **Food processing and UPF framing** | Extent to which food processing, particularly ultra-processed foods (UPFs), is explicitly defined and used as a policy-relevant category | *Are UPFs explicitly defined? Is processing used as a classification framework? Is it linked to health outcomes or policy action?* | Explicit recognition of UPFs as a category of concern; explicitly linked to diet quality and emerging evidence on health outcomes | No explicit UPF terminology; focus on limiting foods high in sugar, salt, and fats; emphasis on fresh and minimally processed foods | **Explicit framing (U.S.) vs Implicit framing (Italy)** | **U.S. guidelines adopt processing as an explicit structural dimension; Italian guidelines rely on nutrient profiling and dietary patterns** |
| **Alcohol risk communication** | Extent to which alcohol-related health risks are explicitly communicated and operationalized in recommendations | *Is alcohol discouraged, limited, or excluded? Is risk explicitly stated? Are thresholds defined?* | Recommendation to limit alcohol consumption; moderate intake remains permissible | Explicit statement that alcohol is harmful; no protective role recognized; discouragement emphasized | **Moderate risk framing (U.S.) vs Explicit risk framing (Italy)** | **Italian guidelines provide clearer precautionary messaging; U.S. guidelines maintain moderated, context-dependent recommendations** |
| **Sustainability integration** | Extent to which environmental sustainability is incorporated into dietary guidance and translated into recommendations | *Are environmental impacts included? Are sustainability criteria operationalized (e.g., local food, waste reduction)?* | Sustainability excluded from formal guideline scope | Explicit integration of sustainability (local and seasonal foods, reduced meat consumption, food waste reduction) | **Absent (U.S.) vs Integrated (Italy)** | **Italian guidelines incorporate sustainability within dietary recommendations; U.S. guidelines remain primarily focused on health outcomes** |
| **Cultural anchoring and communication strategy** | Extent to which dietary guidance is linked to cultural practices and traditions versus generalized health messaging | *Are recommendations culturally embedded? Is communication linked to traditional dietary patterns or generalized?* | Generalized, health-centered communication adapted to diverse populations | Strong Mediterranean cultural framing (tradition, conviviality, seasonal foods) | **Low cultural anchoring (U.S.) vs High cultural anchoring (Italy)** | **Italian guidelines are characterized by strong cultural anchoring; U.S. guidelines prioritize generalizability across diverse populations** |
| **Implementation and scalability** | Extent to which guidelines are supported by institutional mechanisms enabling implementation at scale | *Are guidelines operationalized through programs or systems? Are measurable targets required?* | Direct integration into federal programs; requires operational targets compatible with procurement systems | Implemented through education, communication campaigns, and public health initiatives | **High structural implementation (U.S.) vs Moderate communication-based implementation (Italy)** | **U.S. guidelines are structurally embedded within institutional systems; Italian guidelines rely more on communication and education strategies** |
